# Trends in Coverage and Content of Maternal and Neonatal Care in Bamako, Mali

**DOI:** 10.1007/s11524-024-00931-2

**Published:** 2024-11-22

**Authors:** Mariam Traoré, Djeneba Coulibaly, Fatou Diawara, Ibrahim Terera, Haoua Dembelé, Ababacar I. Maiga, Akory Ag Iknane, Abdoulaye Maïga, Agbessi Amouzou

**Affiliations:** 1 National Institute of Public Health of Mali, Bamako, Mali; 2 Faculty of Pharmacy, University of Sciences Techniques and Technologies of Bamako, Bamako, Mali; 3 Department of International Health, Johns Hopkins University Bloomberg School of Public Health, Baltimore, MD USA

**Keywords:** Inequalities, Care, Mother, Newborn, Bamako

## Abstract

Coverage levels of maternal and neonatal health services in Mali’s major cities vary due to the combined effect of several factors, including poverty and migration to urban centers. This worsened from 2012 due to the security crisis. We conducted an analysis of the trends and differences in several indicators of maternal and neonatal care coverage in Bamako using secondary data from Mali’s Demographic and Health Surveys from 2001 to 2018. Our results highlighted differential access to antenatal and childbirth care for non-poor and non-migrant women compared to their counterparts categorized as poor and migrant. The gaps were much larger depending on migration status (i.e., number of years since resettling in Bamako) and even tended to increase over time. This was particularly the case regarding the number of antenatal visits (ANC 4+), with differences according to poverty level at 7 percentage points in 2001 and 8.3 percentage points in 2018. Migration status showed even larger gaps to the disadvantage of migrant women of 13.4 percentage points (2006) and 24.4 percentage points (2018). There is a higher proportion of cesarean section among non-poor women. The results suggested an opposite pattern for postnatal care of newborns, with a difference of 6.8 percentage points of coverage in favor of the poor in 2018. The high coverage of maternal and newborn health interventions in Bamako city conceals intra-urban disparities to the detriment of poor migrant women and those who recently migrated to the city, partly due to the conflicts and security issues. A redefinition of health programs to include such targets would be desirable from an equity perspective.

## Introduction

Despite recent progress, maternal and neonatal mortality remains a major public health problem worldwide. Approximately 287,000 women died during pregnancy and childbirth around the world in 2020 [1]. Sub-Saharan Africa and South Asia are the two regions that collectively accounted for about 87% of maternal deaths [1]. The worldwide neonatal mortality rate was 17 per 1000 live births in 2022 [2].

Antenatal care (ANC), skilled delivery, and postnatal care (PNC) are some of the major interventions that have contributed significantly to reducing maternal mortality worldwide [3].

In Mali, according to the 2018 Demographic and Health Survey (DHS), only about 43% of pregnant women had at least four antenatal care visits, which is the internationally recommended standard. Other 2018 survey findings included 67% coverage for institutional deliveries and 56% of new mothers receiving postnatal care in the first 2 days after delivery [4]. Such statistics at the national level obscure inequalities between rural and urban areas [4, 5].

This study focuses on the Bamako district, which is considered an entirely urban area in the country. The exodus from rural areas and other cities to Bamako has intensified over the past 10 years due to the socio-political and security situation in the country. From 2009 to 2020, the population of the city of Bamako almost doubled with an urbanization rate that rose from 22.5 to 30,4% [6, 7]. From the point of view of economic well-being, the percentage of poor people in Bamako declined between 2012 and 2018. That trend contributed to and reflects the fact that during this period, almost all of the city’s population, Bamako (between 83 and 86%) was classified in the two richest quintiles (Q4 et Q5) [6].

Substantial research has focused on inequalities in maternal and neonatal health care, especially in developing countries [5].

However, the particularity of the urban environment in terms of inequalities has remained relatively unexplored, with far more limited research examining intra-urban or rural differences that may be accentuated by factors such as poverty or migration (rural exodus). This situation is especially relevant in the case of the city of Bamako. Poverty and migration are factors that could thus influence trends in maternal and neonatal care indicators and their quality and be major obstacles to reducing maternal and neonatal mortality. This study was part of an effort to better understand such factors and their impacts. Its objective was to analyze the trends and differences in coverage and content of maternal and neonatal care in Bamako, Mali.

## Methods

### Setting

Bamako, the administrative, political and economic capital of Mali, covers an area of 267 square kilometers (104 square miles) [8]. Its population is estimated at 4,227,569 inhabitants in 2022 [7], i.e., a density of 15,833 inhabitants/square kilometers. The city is divided into 6 communes and 67 wards. In 2014, Bamako had 6 referral health centers and 58 community health centers [8].

Since 2012, Mali has been exposed to conflict and insecurity situations that have caused significant population displacement across the national territory. According to the National Directorate of Social Development, commune VI of Bamako has a site for internally displaced persons. This site has the characteristics of a shanty town with straw buildings, little water supply, no health center, or school of their own.

**Map 1:**
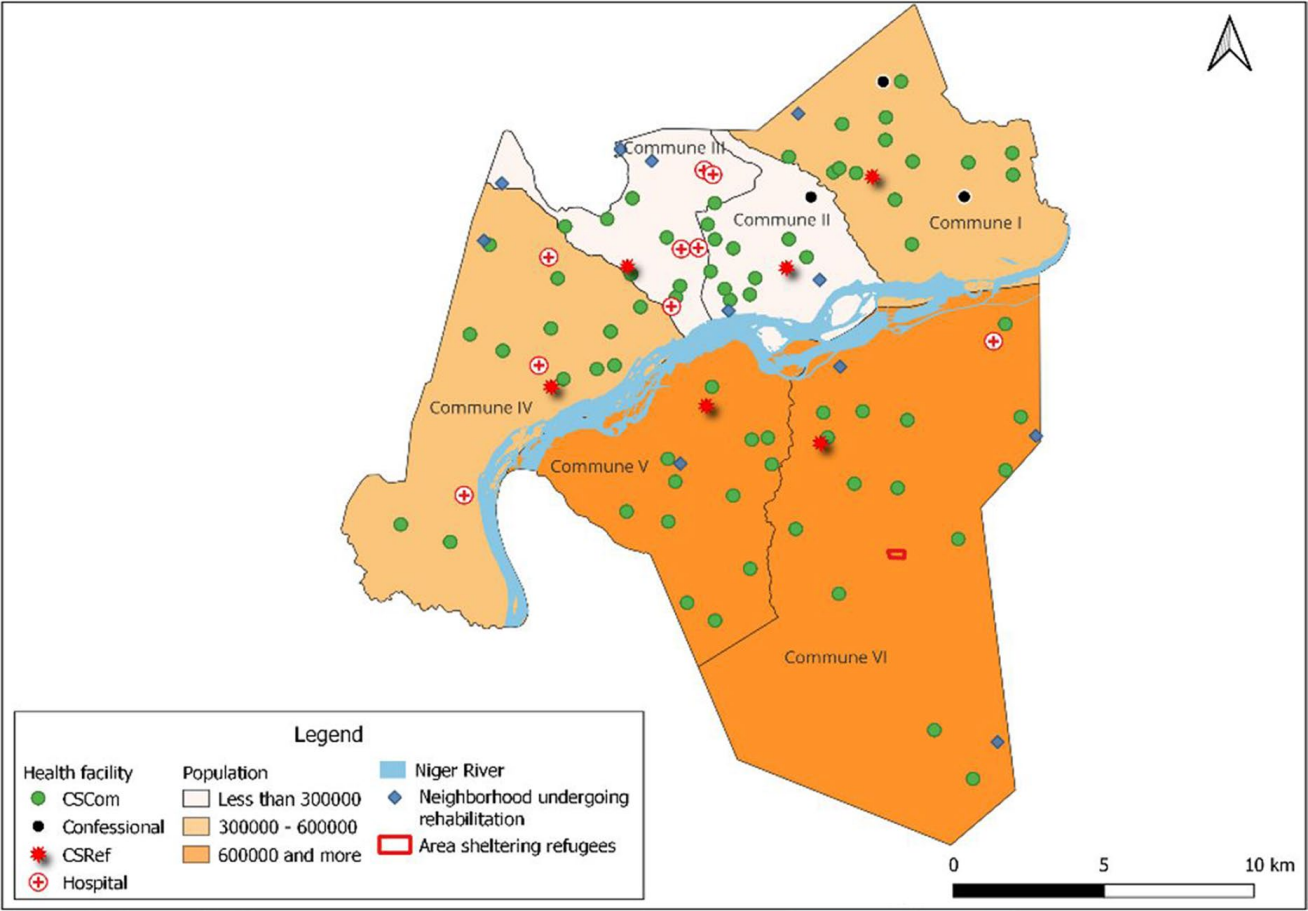
Administrative and health divisions of Bamako in 2018. CSCom, community health center; CSRef, referral health center. Source: INSP 2023

Map 1, which is based on data from 2018, shows that communes V and VI are the most populated and are located on the right bank of the Niger River. According to the urban planning and housing services of the various communes, the district of Bamako has 10 wards in the process of urban rehabilitation as of 2022.

### Data Sources

An analysis of secondary data from the Demographic and Health Surveys (DHS) from 2001 to 2018 was conducted. DHS are national stratified, and two-degree area surveys are designed to provide information on Mali’s population in the areas of demographics, health, and nutrition. Databases available on the DHS program served as data sources, as well as reports from DHS surveys.

### Measures

To achieve its objectives, the study aimed to analyze the coverage of maternal and child health interventions and the quality of these interventions. This required secondary analysis concerning variables of interest including delivery in a health center, cesarean section (C-section), antenatal care (ANC), and postnatal care (PNC). In order to highlight socio-economic inequalities according to the level of poverty and the strong growth of migratory flows towards the city of Bamako during the study period 2001–2018, the explanatory variables were the wealth index and the migratory status of women.

We defined migration status around a point 2 years prior to each survey: (i) former migrant (living more than 2 years in Bamako), (ii) recent migrant (living in Bamako less than 2 years), and (iii) resident (has always lived in Bamako).

The variables included for the calculation of the quality of postnatal consultation (PNCq) of the newborn were (1) weighing the newborn, (2) observing breastfeeding, (3) advice on breastfeeding, (4) advice on danger signs, (5) taking the newborn’s body temperature, and (6) examining the newborn’s umbilical cord.

### Methods of Analysis

The wealth index, calculated by the DHS, is a relative measure of poverty for the entire national sample. The wealth index for the city of Bamako alone was recalculated for the purposes of this study. This recalculation was done in accordance with the DHS methodology and by targeting the variables relevant to the urban context. Databases available on the DHS program served as a data source, as well as reports from DHS surveys: https://dhsprogram.com/topics/wealth-index/ and https://dhsprogram.com/topics/wealth-index/Wealth-Index-Construction.cfm.

The wealth index was divided into five equal categories, each representing 20% of the population. To establish two modalities only, the poorest and the next poorest quintiles (40% of the total) were combined into one “poor” category and the median, the richest, and the next richest quintiles (the remaining 60%) into “non-poor” category.

Concerning the migrant status, to establish only two modalities of this variable, we combined former migrant and resident into one “non-migrant” category while considering those who have lived less than 2 years in Bamako to be “migrants.” These groupings were done to accommodate with sample size issues.

The quality of antenatal care is defined by the ANCq indicator. It takes into account the contact and content of antenatal care, which have been calculated based on a methodology validated by studies on the development and validation of a score using national health surveys in low- and middle-income countries [9] and on inequalities in the coverage and quality of antenatal care [10]. Thus, the ANCq score varies from zero, for women with no antenatal care, to 10 points for women with the best results for each item [10]. The ANCq scores were then categorized into a binary variable corresponding to 1 for those with a score between 8 and 10 points and 0 for those with a score below 8.

We calculated the postnatal content of care variable, which we used as a proxy for quality of neonatal care (PNCq), using the same methodology as ANCq. We assessed quality by taking into account the total number of actions received by the newborn. The PNCq score obtained was transformed into a binary variable taking 1 as a value for newborns with 5 or more actions and 0 as a value for those with fewer than five actions.

We then compared proportions of quality indicators (ANCq and PNCq) to poverty status (poor and non-poor) and migration status (migrant and non-migrant).

To assess the trends of maternal and neonatal care indicators, we presented trends from 2001 to 2018 from the reports of DHS through confidence interval (CI) bar charts. We analyzed the 95% CIs to see if they overlapped to assess the significance of the differences in coverage between the indicators for the poor and non-poor and migrants and non-migrants. We analyzed data using software stata 14.2, Excel was used for graphical representations. In order to conduct the analysis, we compared the indicator coverage using proportions. Key limitations included postnatal care data being available only for 2012 and 2018 and migration data being available only for 2006 and 2018.

## Results

### Level of Coverage and Quality of Antenatal and Postnatal Care in 2018

**Fig. 1:**
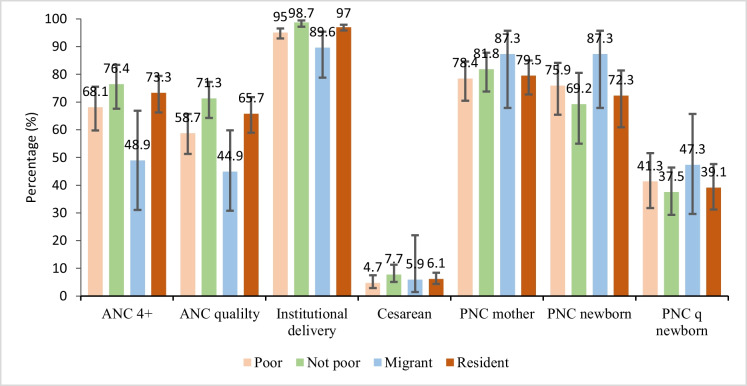
Coverage and content of maternal and neonatal care according to the status of poverty and migration in Bamako in 2018

Figure 1 shows the level of coverage of the interventions studied in 2018. A notable finding is that for all interventions, except for postnatal and its quality in newborns, the level of coverage among non-poor women exceeded that of poor women. The differences observed between poor and non-poor were not significant for the achievement of 4 ANC or more, for the quality of ANC (ANCq), for cesarean section and postnatal care coverage for the mother (Fig. 1).

For institutional delivery, the gap of 3.7 percentage points between poor and non-poor was significant with 95% CI [92.9–96.3] among the poor and [97.2–99.4] among the non-poor. Regarding migration status, there were insignificant differences in favor of women who had non-migrant (resident) status compared to migrants in the achievement of at least four antenatal care visits, as well as in the quality of antenatal care. This observation is also valid for cesarean section among non-migrants even if the difference is very minimal, i.e., 0.2 percentage points (Fig. 1).

The difference was significant for delivery in a health center, at 24.4 points with 95% CI: [95.8–97.9] for non-migrants and [78.8–95.4] for migrants. Non-migrant women gave birth more in health centers, and there was significant inequality between the two.

Analysis of the quality of postnatal care among newborns (PNCq) showed that there was a gap of 8.2 percentage points in favor of migrants. Analysis of confidence intervals showed that these differences were not significant, at 95% CI [29.6–65.7] for migrants and [31.2–47.6] for non-migrants.

### Changes in Coverage and Content of Antenatal, Delivery and Postnatal care

#### Trends in Coverage and Content of Maternal and Neonatal Care by Poverty Status in Bamako from 2001 to 2018

Analysis of trends from 2001 to 2018 showed an increase in coverage for all maternal and newborn health interventions. Achievement of ANC 4+ increased among the poor from 66% (95% CI [61.5–70.1]) in 2001 to 68.1% (95% CI [59.7–75.5]) in 2018, and among the non-poor, it varied from 72.9% (95% CI [67.6–77.6) in 2001 to 76.4% (95% CI [67.6–83. 4]) in 2018 (Table 1 in the Appendix).

A gap has always existed between the poor and the non-poor in terms of coverage of the indicators. However, the gaps were not always significant from 1 year to the next. For ANC 4+ , in 2006, the gap was in favor of the non-poor and increased significantly by 12.8 points (95% CI: [55.9–67.7] for the poor and [68.9–79.9] for the non-poor) (Table 1 in the Appendix). The gap of 17.5 percentage points observed in 2012 was also significant with 95% CI: [58.2–68.1] for the poor and [76.5–84.5] for the non-poor. In 2018, the gap was 8.3 percentage points but was not significant according to the analysis of confidence intervals (Table 1 in the Appendix).

**Fig. 2:**
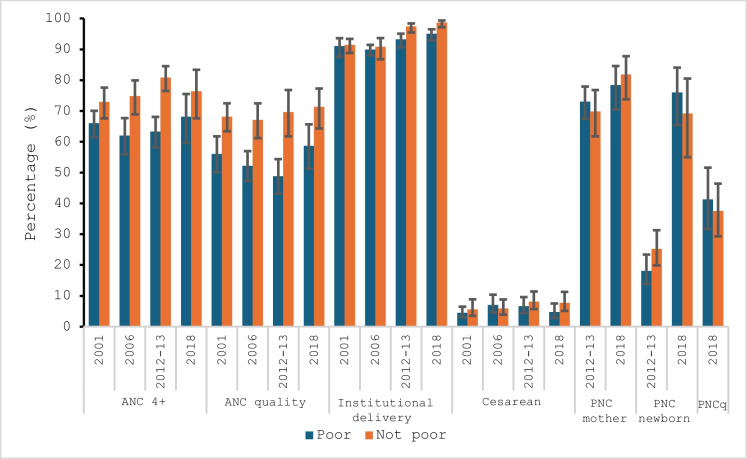
Trends in coverage and content of maternal and neonatal care by poverty status from 2001 to 2018 in Bamako, Mali

The proportion of women for whom ANC was of quality (ANCq) varied among the poor, going from 56% in 2001 to 58.7% in 2018 with a minimum of 48.8% in 2012. Among the non-poor, coverage has almost increased (Fig. 2). Analysis of confidence intervals showed that the differences increased in favor of the non-poor from 14.9 points in 2006 (95% CI [43.7–57.0] poor; [61.2–72.5] non-poor), to 20.8 points in 2012 (95% CI: [43.2–54.4] poor and [64.0–74.7] non-poor) (Table 1 in the Appendix). The gap of 12.6 points observed in 2018 was not significant (Table 1 in Appendix).

Concerning institutional childbirth, overall, the level of coverage has increased slightly over time for the two classes except for the year 2006 where the levels did not reach those of the other years (Fig. 2). We note a variation in the gaps ranging from 0.4 percentage points in 2001 to 3.7 points in 2018 with a maximum of 4.2 points in 2012. The gaps observed in 2012 and 2018 were significant in favor of the non-poor (Table1 in Appendix).

In addition, there were overlaps in confidence intervals between the proportions of poor and non-poor women for the indicators of C-sections, mother and newborn postnatal care, and the quality of newborn postnatal care (PNCq) over the duration of the study (Fig. 2). There were differences in favor of the non-poor in most cases, but these differences were not significant. Thus, no significant inequality was observed between poor and non-poor people in terms of C-section, postnatal care, and its quality over time (Fig. 2).

#### Trends in Coverage and Content of Maternal and Neonatal Care by Migration Status in Bamako from 2001 to 2018

**Fig. 3:**
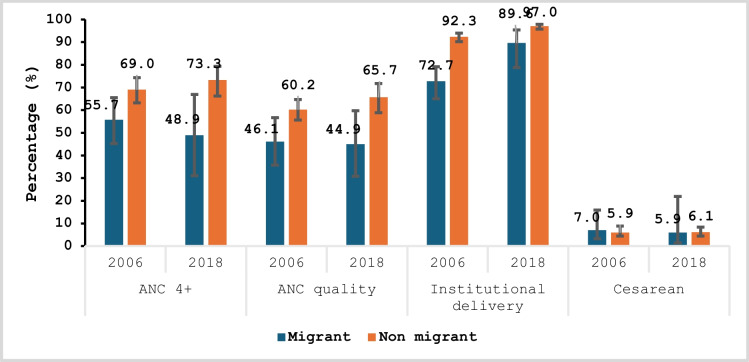
Trends and inequalities in coverage and quality of maternal care by migration status in 2006 and 2018 in Bamako

Figure 3 shows that in terms of migration status, ANC 4+ and ANCq coverage had similar trends. From 2006 to 2018, there was a decrease in coverage among migrants (55.7 to 48.9% for ANC 4+ and 46.1 to 44.9% for ANCq) and an increase among non-migrants (69.0 to 73.3% for ANC 4+ and 60.2 to 65.7% for ANCq). Except for C-section, where no significant difference was observed, the differences were in favor of non-migrants regarding ANC 4+ , ANCq, and delivery in a health center. The analysis of CI revealed that these differences were not significant except for the institutional delivery of 2018.

For ANC 4+ , the gap ranged from 13.3 percentage points in 2006 to 24.4 points in 2018; for ANCq, the gap was 14.1 percentage points in 2006 and 20.8 points in 2018.

On the other hand, the differences decreased significantly for institutional delivery, ranging from 19.6 percentage points (2006) to 7.4 points (2018) in favor of non-migrants (Table 1 Appendix). Key overall observations are that non-migrants give birth more in health facilities, and the inequality between migrant and non-migrant was significant.

**Fig. 4:**
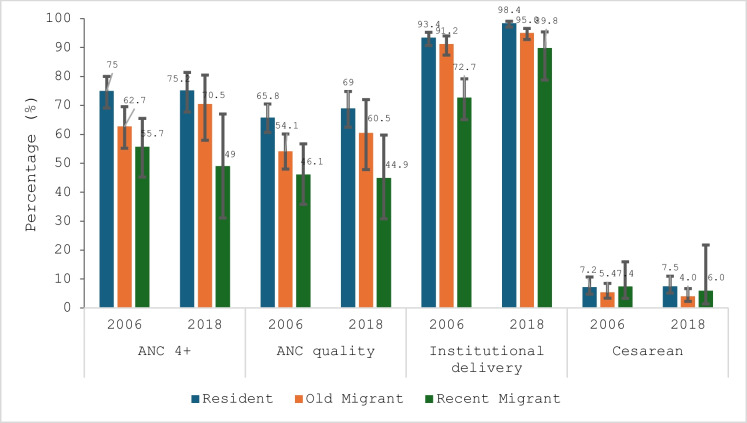
Trends and inequalities in coverage and quality of maternal care by duration of migration in 2006 and 2018 in Bamako

Figure 4 presents the results for migration duration. Analysis of the coverage of interventions shows that in 2006 and 2018, the coverage in the realization of ANC 4+ and ANCq among former migrants (those living in Bamako for at least 2 years) increased from 62.7 to 70.4% and from 54.1 to 60.5% respectively. However, over the same period, coverage for recent migrants (living in Bamako less than 2 years) decreased from 55.7 to 48.9% and from 46.1 to 44.9% for ANC 4+ and ANCq, respectively. These increases and decreases in coverage are not significant given the overlap in their confidence intervals.

As also illustrated in Fig. 4, there is no significant difference between non-migrants and former migrants in the coverage of the four indicators. However, the differences between non-migrants and recent migrants are statistically significant for two of them (ANC 4+ and ANCq in 2018). These results suggest that in 2018, recent migrants, compared to non-migrants, achieved less ANC 4+ and less than half received the full content of quality antenatal care.

## Discussion

Our study analyzed trends in coverage and quality of maternal and neonatal health interventions in Bamako. In general, regardless of poverty or migration status, the level of coverage of the interventions studied increased over the period studied (2001 through 2018).

The rise in coverage could be explained by the government’s willingness to implement health reforms that included (i) a notable increase in the number of functional community health centers, from 39 in 1999 to 58 in 2018 [11]; (ii) the implementation of several components of emergency obstetric and neonatal care (EmONC) packages that aim to reduce maternal and neonatal mortality [12]; (iii) essential newborn care (ENC) training and services [13]; (iv) free access to C-section and some components of antenatal care (e.g., distribution of insecticide-treated nets, malaria chemoprophylaxis with sulfadoxine/pyrimethamine, anti-tetanus vaccination) [12]; and (v) strengthened social protection policies that included compulsory health insurance for many formal workers and their families [14].

Comparable results were observed in the studies of Hajizadeh M et al. and Alam N et al. who found that similar health reforms correlated with increased use of maternal health services in countries including Bangladesh, Ethiopia, Madagascar, and Uganda [15, 16].

Despite the progress seen in the use of maternal health services and the government’s efforts to ensure equity and quality of care adopted in the 10-year Social Health Development Plan 2014–2023, declines occurred in poor women’s use of antenatal care services in Bamako from 2006 to 2012 and in institutional delivery services between 2012 and 2018. Our results agree with those of Ganle J K et al. in Ghana and Boutayeb A et al. in Morocco and Egypt, where women in the lowest income quintile were found to use antenatal and childbirth services less than in the highest income quintile [17, 18]. Matthews Z et al. in 2010 found that in some countries, such as Cambodia and Nepal, birth rates in health facilities of over 80% for urban non-poor women coexist with rates of about 20% for urban women in the poorest quintile [19].

These inequalities observed between the poor and the non-poor were significant for the achievement of ANC 4+ in 2006 with 12.8 percentage points and in 2012 with 17.5 percentage points in favor of the non-poor. Therefore, the non-poor achieved more ANC 4+ in 2006 and 2012, and there was a significant inequality. The quality of ANC has increased over time among the non-poor, and there was significant inequality in their favor in 2006 and 2012. Similar results are observed in 2008 in Nigeria by Obiyan M O et al. who find an increase in inequalities in the use of ANC from 0.467 in 1990 to 0.621 in 2015 [20].

Although the non-poor in Bamako gave birth in a health center more often than the poor across the entire study period, there was a significant reduction in the gap between 2012 and 2018.

The policy of bringing health centers closer to the population could have contributed to this reduction in inequality because in 2012, 93% of the population of Bamako had access to a health center within a radius of 15 km compared to 100% in 2018 [11]. Our results are similar to those obtained by Ravit M et al. which showed a significant reduction in inequalities in access to institutional childbirth between 2001 and 2012 in Mali and Benin [21].

However, Alam N et al. found that absolute inequalities between poor and non-poor in institutional delivery increased in all countries studied except Zambia where absolute differences decreased by 0.075 points (CI: [− 0.115 to − 0.035]) between the first and last surveys [16].

There was no significant inequality between the poor and the non-poor in cesarean section services, PNC, and its quality over time.

Regarding the duration of migration, non-migrants and former migrants had higher coverage for care indicators such as childbirth in a health center, ANC 4+ , and ANCq compared to recent migrants. The differences were significant between non-migrants and recent migrants for ANC 4+ in 2018, which could be explained by the influence of duration in residential environment. That explanation suggests that former migrants tend to adopt the same behavior as non-migrants in the use of antenatal care services. Indeed, indicator coverage was observed to have increased among former migrants while it declined among recent migrants from 2006 to 2018.

Among migrants, ANCq coverage declined slightly, from 46.1% (95% CI: [35.8–56.7]) in 2006 to 44.9% (95% CI: [30.8–59.8]) in 2018, but the differences were not significant over time. In addition to this sawtooth trend, the differences between recent migrants and non-migrants increased significantly in 2018.

This analysis suggests that interventions put in place to improve coverage were not having notable impacts on quality. The goal of antenatal care is to allow early detection of fetal abnormalities and complications in pregnant women. For these problems to be detected as widely as possible, antenatal care must start early to reach and ideally exceed the four recommended visits before birth and also the maximum number of women must benefit from more of the recommended actions at each session. Being poor and a migrant can be obstacles to the achievement of both of these goals. For the poor, even if some care services are free, there is nevertheless a minimum of costs that are generated by their use (e.g., the need to spend money out-of-pocket on a consultation ticket, antenatal care booklet, medicines).

For migrants, not being sufficiently informed about the existence of these services or their availability can be an obstacle. In addition, for migrants, there are sometimes other obstacles such as lack of awareness of the importance of these services and various socio-cultural reasons.

Although these elements could explain some of the differences observed in coverage, other factors such as those related to the training and behavior of the caregivers must be explored in order to understand the differences observed in the quality of antenatal care. The potential major impact of that factor is supported by Benova L et al. in 2018, who found that a focus on the number of antenatal visits may have been at the expense of effective (i.e., quality) coverage, because in the results, the countries with the highest overall coverage did not have in the content analysis the high proportions of recommended actions carried out [22]. Similar results are reported by Siddique A B et al. in 2018 in Bangladesh in low- and limited-resource countries that conclude that many low-resource countries have achieved high levels of antenatal care coverage while quality levels were much lower and inequitable [23].

In 2018, newborns from poor and migrant categories had more contact with postnatal care services and also received better quality care compared to non-migrants (residents) and non-poor, but the differences were not significant.

In general, the differences observed for C-section were exceedingly insignificant compared with the other indicators. This could be explained by the government’s policy of free C-sections for all women regardless of their social status.

## Limits

This study has a few limitations. Sample size issues caused large confidence intervals affecting statistical tests and comparisons. Also, the retrospective nature of the surveys did not provide sufficient data on postnatal consultations.

## Conclusion

In general, regardless of the status of poverty and migration, the level of coverage of the maternal and newborn health interventions studied increased over time. The trends analysis by level of poverty revealed that the level of coverage of interventions increased among both those classified as poor and non-poor. In addition to this increase in coverage, there were inequalities between poor and non-poor in the use of these different services. These inequalities were not significant for caesarean section, PNC, and the quality of PNC over time. Regarding migration status, the differences were in favor of non-migrants; they increased for ANC 4+ and ANCq and decreased significantly for institutional delivery between 2006 and 2018. For cesarean section, in 2006, the difference was in favor of migrants, and in 2018, the trend reversed in favor of non-migrants. Coverage of ANC 4+ has increased over time, however, the quality of ANC has not improved among the poor (from 2001 to 2012) and among recent migrants (2006 to 2018).

The existence and persistence of such inequalities support closer reflection on and consideration of the relative weak ability of programs aimed at reducing maternal and infant mortality to affect the most disadvantaged. It is therefore necessary for policy makers and stakeholders involved in the implementation of these interventions to closely monitor quality and equity for disadvantaged targets in the urban context.

## Figures and Tables

**Table 1:**
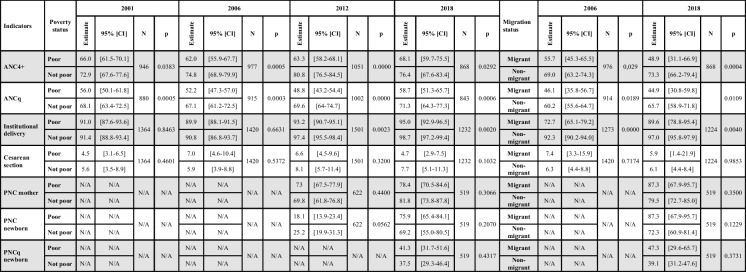
Maternal and neonatal health care indicators by poverty and migration status from 2001 to 2018 in Bamako, Mali

*N/A* not available, *ANC* 4+antenatal care four or more visits, *ANCq* antenatal care quality, *PNC* postnatal care, *PNCq* postnatal care quality

## Data Availability

The study utilizes the Mali Demographic and Health Survey (DHS) data, which is publicly available for free download after registration as a DHS data user.
